# High polymorphism in *MHC-DRB* genes in golden snub-nosed monkeys reveals balancing selection in small, isolated populations

**DOI:** 10.1186/s12862-018-1148-7

**Published:** 2018-03-13

**Authors:** Pei Zhang, Kang Huang, Bingyi Zhang, Derek W. Dunn, Dan Chen, Fan Li, Xiaoguang Qi, Songtao Guo, Baoguo Li

**Affiliations:** 1 Shaanxi Key Laboratory for Animal Conservation, College of Life Sciences, Northwest University, Xi’an, China; 2 Middle School Affiliated to Northwest University, Xi’an, China; 3 Xi’an Branch of Chinese Academy of Science, Xi’an, China

**Keywords:** *MHC*, *Rhinopithecus roxellana*, Genetic diversity, Balancing selection

## Abstract

**Background:**

Maintaining variation in immune genes, such as those of the *major histocompatibility complex* (*MHC*), is important for individuals in small, isolated populations to resist pathogens and parasites. The golden snub-nosed monkey (*Rhinopithecus roxellana*), an endangered primate endemic to China, has experienced a rapid reduction in numbers and severe population fragmentation over recent years. For this study, we measured the *DRB* diversity among 122 monkeys from three populations in the Qinling Mountains, and estimated the relative importance of different agents of selection in maintaining variation of *DRB* genes.

**Results:**

We identified a total of 19 *DRB* sequences, in which five alleles were novel. We found high *DRB* variation in *R. roxellana* and three branches of evidence suggesting that balancing selection has contributed to maintaining *MHC* polymorphism over the long term in this species: i) different patterns of both genetic diversity and population differentiation were detected at *MHC* and neutral markers; ii) an excess of non-synonymous substitutions compared to synonymous substitutions at antigen binding sites, and maximum-likelihood-based random-site models, showed significant positive selection; and iii) phylogenetic analyses revealed a pattern of trans-species evolution for *DRB* genes.

**Conclusions:**

High levels of *DRB* diversity in these *R. roxellana* populations may reflect strong selection pressure in this species. Patterns of genetic diversity and population differentiation, positive selection, as well as trans-species evolution, suggest that pathogen-mediated balancing selection has contributed to maintaining *MHC* polymorphism in *R. roxellana* over the long term. This study furthers our understanding of the role pathogen-mediated balancing selection has in maintaining variation in *MHC* genes in small and fragmented populations of free-ranging vertebrates.

**Electronic supplementary material:**

The online version of this article (10.1186/s12862-018-1148-7) contains supplementary material, which is available to authorized users.

## Background

The *major histocompatibility complex* (*MHC*) gene family is one of the most polymorphic gene regions yet found in any vertebrate genome [1]. This multigene family plays a central role in the immune systems of many vertebrates by first recognizing foreign antigens and then binding and presenting them to T cells, thus triggering an appropriate immune response [2, 3]. Specifically, *MHC class I* and *II* genes encode cell surface glycoproteins that bind intracellular (such as viruses) and extracellular foreign peptides (such as bacteria and parasites), respectively [4, 5]. The *class II* gene series *DP*, *DQ*, and *DR* encode heterodimeric proteins each with α and β chains. The *DR* β (*DRB*) genes, especially exon 2, which encodes functionally important antigen binding sites (ABS), has been extensively studied in mammals [6–8]. Variation in residues within the ABS of different *MHC* alleles defines the range of antigens that the immune system can identify and fight-off [9]. Thus, pathogen-mediated balancing selection is a major agent shaping *MHC* polymorphism as a consequence of an arms-race between pathogens and the host’s immune system [9].

Three forms of balancing selection that may maintain variation in the *MHC* have been recognized: heterozygote advantage, negative frequency dependent selection, and fluctuating selection [1, 10, 11]. All three forms of balancing selection may act simultaneously in maintaining *MHC* variation.

Three methods are often used to detect balancing selection. First, natural selection can be estimated via calculating the selection parameter *ω* (*d*_N_/*d*_S_, the rate of non-synonymous substitutions/synonymous substitutions) and is the most common method used [11, 12]. According to the neutral selection theory, *ω* should not significantly deviate from one [13]. When *ω* is significantly greater than one this indicates positive selection, and in the case of *MHC* genes, balancing selection due to host/pathogen coevolution [14]. Conversely, when *ω* is significantly less than one, this indicates negative/purifying selection [15]. Second, trans-species evolution is another common method used to identify historical balancing selection on *MHC* genes, in which the same advantageous *MHC* alleles are conserved across distinct evolutionary units in spite of differentiating evolutionary processes [16]. Third, different patterns of genetic diversity and population differentiation for genes under selection compared to neutral genes, can also indicate the presence of balancing selection [17].

In practice, the role of balancing selection in maintaining adaptive variation in the *MHC* is still unclear, because various evolutionary factors can affect *MHC* variation and may mask any effects of balancing selection [18]. More specifically, in small and/or fragmented populations, genetic drift is likely to reduce *MHC* diversity [19, 20].

The golden snub-nosed monkey (*Rhinopithecus roxellana*) is an endangered primate endemic to China. Although once widespread in China, wild *R. roxellana* populations now only occur in fragmented populations in Sichuan, Gansu, Hubei and Shaanxi provinces. This study was conducted in the Qinling Mountains, Shaanxi province, the major east-west mountain range of China. These mountains provide a natural boundary between northern and southern China, and support much biodiversity. Within the Qinling Mountains, there are 39 known *R. roxellana* populations comprising a total of ~ 4000 individuals [21]. The average population size is about 100 individuals. Each population concluded a breeding band, an all-male band and several solitary males [22]. Normally, males are able to migrate between neighboring populations (< 5 km). While females often stay in their natal population, they can also migrate to neighboring populations via seasonal fission-fussion [22]. However, over the past few decades, suitable habitat for this species has decreased rapidly in both quality and quantity, and has become fragmented due to commercial logging, and the building of roads or other infrastructures [21]. The effects of fragmentation have also been exacerbated through human activities such as increased tourism, hunting, agricultural expansion, herb collecting, and firewood collection [21]. This has resulted in a reduction of the total *R. roxellana* population by more than 50% over the past 40 years, with *R. roxellana* being classified as endangered since 2008 by the IUCN [23].

Small and/or fragmented populations will experience reduced genetic variation due to inbreeding, restricted gene flow and genetic drift [24]. The degree of genetic variation is thought to facilitate the potential of small and/or fragmented populations to adapt to environmental change. Maintaining genetic variation in such populations is thus a critical component in appropriate conservation strategies for endangered species such as *R. roxellana* [25].

Previous studies of *R. roxellana* populations using neutral markers (such as mitochondrial D-loops and microsatellites) to assess genetic diversity have provided much information on phylogenetic relationships and demographic history [26, 27]. For example, microsatellite analysis revealed relatively high levels of both genetic diversity and population subdivision of Qinling Mountains *R. roxellana* [28]. Unfortunately, neutral markers cannot provide direct information associated with the ability and capacity of hosts resisting continuously evolving parasites and pathogens in the natural environment. To date, the adaptive nature of *MHC* variation under pathogen-mediated coevolution in different Qinling Mountains *R. roxellana* populations is unknown.

In this study, we i) measure genetic variation of *MHC* genes and microsatellite loci in three Qinling Mountains *R. roxellana* populations, ii) identify different agents of selection (including positive selection and trans-species evolution) and the roles they may play in maintaining variation in *MHC* diversity, and iii) evaluate the potential for balancing selection for *MHC* genes in *R. roxellana* populations. This study furthers our understanding of pathogen-mediated balancing selection at *MHC* genes in small and/or fragmented populations of free-ranging vertebrates.

## Methods

### Sampling and DNA extraction

**Fig. 1:**
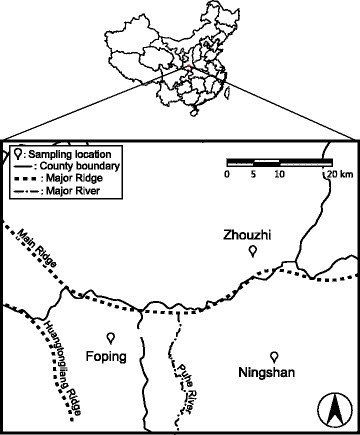
Distribution of the three *R. roxellana* populations used for this study. The range of coordinates were 107.8°-108.5°E, 33.5°-34.0°N, UTM projection

A total of 122 wild *R. roxellana* samples were collected from three populations in the Qinling Mountains, including 32 hair samples from Foping (FP), 36 fecal samples from Ningshan (NS), and 54 hair samples from Zhouzhi (ZZ) (Table 1). These populations were located in three officially protected nature reserves in three counties (Fig. 1). The FP and NS populations were located on the southern slope of the Qinling Mountains, whereas the ZZ population was located on the northern slope. The three study populations belong to three different reserves (Fig. 1). Long distances and habitat fragmentation prevent monkeys from migrating between these three populations [28].

**Table 1:** The location, population size, number of samples and sampling time of each study population

Population	Reserve	Location	Size	Samples^a^	Sampling time
Foping (FP)	FNNR	33.68°N, 107.99°E	70	32/0	November 2014
Ningshan (NS)	NNR	33.66°N, 108.35°E	100	0/36	January 2015
Zhouzhi (ZZ)	ZNNR	33.79°N, 108.26°E	110	54/0	December 2014

^a^Numbers in the front of and behind the slash denoted the hair and feces samples collected, respectively. Size, estimated population size; FNNR, Foping National Nature Reserve; NNR, Ningshan Nature Reserve; ZNNR, Zhouzhi National Nature Reserve

Hair samples were collected using a pole with glue on its end. Each sample was stored individually at room temperature in a labeled envelope in a dryer containing desiccant granules. Fresh fecal samples were stored in DMSO salt solution (DETs: 20% DMSO, 0.25 M sodium-EDTA, 100 mM Tris-HCl, pH 7.5, and NaCl to saturation) at − 20 °C. Both individual identification and sample collections complied with the animal welfare laws and constitutions of China. DNA was extracted from hair samples according to a Chelex protocol (Chelex 100, Bio-Rad) [29]. Fecal DNA was extracted using QIAamp DNA Stool Mini Kits (Qiagen, Germany). Individuals were identified via microsatellite profiles with 19 loci to exclude repeated samples from a same individual [28]. During the extraction and subsequent polymerase chain reaction (PCR), laboratory benches were washed with 75% ethanol. All facilities and disposable plastic-ware used were exposed to UV light for 30 min prior to treatment, preventing contamination by human DNA. For the same purpose, negative controls were used for each PCR reaction.

### Molecular techniques

Overall genetic diversity was assessed based on 19 microsatellites. All individuals were genotyped at these microsatellites following the previously established methods [28], using the same primers as used in Huang et al. [28]. Adaptive variation was studied in the highly polymorphic *DRB* exon 2 fragments. To amplify the exon 2 of the *DRB* genes in *R. roxellana*, a primer pair (F: 5’-TTCTCAGGAGGCCGCCCGTGTGA-3′; R: 5’-ACCTCGCCGCTGCACTGTGAAGCTC-3′) was used [30]. The length of *DRB* exon 2 is 270 base pairs (bp). The primer pair amplified a 270 bp product, which contains 247 bp of exon 2 and 23 bp of intron 1. PCR was performed in 50 μL of reaction mix containing 10 mM Tris-HCl (pH 8.4), 50 mM KCl, 2.5 mM MgCl_2_, 0.4 μM of forward and reverse primers, 0.2 mM of each dNTP, 1 unit of ExTaq DNA polymerase (Takara, Dalian), and 10–100 ng of genomic DNA. Amplification was carried out in a Veriti™ 96-Well Fast Thermal Cycler (Applied Biosystems, Singapore) under the following conditions: initial denaturation at 94 °C for 5 min, followed by 35 cycles of denaturation at 94 °C for 30 s, annealing at 60 °C for 30 s and extension at 72 °C for 30 s, finishing with a final extension at 72 °C for 10 min.

Amplification products were purified using an AxyPrep™ DNA Gel Extraction Kit (AXYGEN Biosciences) according to the manufacturer’s protocol. Purified PCR product was then ligated into a pMD 18-T Vector (Tarkara, Dalian) and transformed into a DH5α competent cell (Tarkara, Dalian). Twenty positive clones containing inserts from each individual were sequenced in both directions using an ABI-Prism™ 3100 Genetic Analyzer (Applied Biosystems Inc.).

### Data analysis

#### Identification of MHC alleles

All sequences were aligned by clustalx V2.0 [31]. To prevent interference of the PCR amplification artifacts, each new sequence was considered to be an allele if it had been detected in at least two different individuals or in three different PCRs of the same individual. Then, each allele was aligned with Rhro-DRB*01–37 (GenBank accession numbers: JQ863322-JQ863358) [30] and verified with the whole genomic data of *R. roxellana* [32] using the BLAST (Basic Local Alignment Search Tool; https://blast.ncbi.nlm.nih.gov/Blast.cgi) of the NCBI.

Because our sampling was limited to a narrow time window (Table 1) only samples from adult individuals were obtained. Our data therefore did not include any known parent-offspring relationships (parent-offspring duos, or mother-father-offspring trios) to validate the observed individual *DRB* genotypes. We used mhc-typer V1.0 (unpublished, https://github.com/huangkang1987/mhc-typer), a new method for assigning alleles to different loci based on the calculation of likelihood of loci, to assign each allele to a specific locus.

mhc-typer V1.0 uses a simulated annealing algorithm to find the optimal allele configuration, which is defined as a partition of the alleles into several loci [33]. The searching procedure begins from a trivial initial solution (i.e. allele configuration), then randomly mutates to a new solution. The new solution will be accepted according to a ratio that depends on current temperature and the difference in the evaluation value (AIC or BIC) between the new and the current solutions. Both parameters are functions of the likelihood of the genotypes calculated from the allele configuration. If the current temperature is high, the same non-optimal solution will be accepted at a high ratio. Therefore, the searching algorithm randomly ‘walks’ across configurations with high temperatures and becomes increasingly ‘greedy’ (accepts worse configurations at a low probability) as the temperature decreases. The simulated annealing algorithm simulates the annealing of a metal, and begins from a relatively high temperature, and decreases by being repeatedly multiplied by an annealing coefficient of less than one (e.g. 0.99). The annealing cycle is repeated several times to ensure that the optimal solution is found.

#### Genetic variation at MHC and microsatellites

BEV81148*xDRB* sequences were translated into amino acid sequences using mega V7 [34]. Variable sites, parsimony-informative sites, and overall mean genetic distances of nucleotide sequences were derived in mega V7.0 [34]. Deviation from the Hardy-Weinberg equilibrium (HWE) was tested with 100,000 steps of Markov chain using genepop V4.3 [35] for each population. Allelic Richness (*A*_R_) based on minimal sample size and inbreeding coefficient (*F*_IS_) per locus were both calculated using fstat V2.9.3 [36]. Expected heterozygosity (*H*_E_), observed heterozygosity (*H*_O_), polymorphism information content (PIC) and the frequency of null alleles (*P*_null_) were calculated using cervus V3.0 [37]. The effective number of alleles (*A*_E_) per locus and *F*-statistics (*F*_ST_) were both computed using genalex V6.5 [38]. In addition, Tajima’s test was conducted using dnasp V5.10.01 [39]. A positive Tajima’s *D* value means a heterozygous advantage or population reduction, while a negative value represents selection on a specific allele or population expansion [40].

To test whether *MHC* loci were behaving differently from neutral loci, allelic richness (*A*_R_), inbreeding coefficient (*F*_IS_), expected heterozygosity (*H*_E_), observed heterozygosity (*H*_O_), polymorphism information content (PIC) and population differentiation (*F*_ST_) were estimated for both *MHC* and microsatellite loci. We used Mann-Whitney *U* test to compare the *H*_E_ and PIC between these two types of loci. Data were analyzed using spss V22.0 (IBM). All *P*-values are two tailed, and the level significance was 0.05.

#### Phylogenetic analysis

Phylogenetic relationships among *Rhro-DRB* alleles (including data from previous research [30]) (GenBank accession numbers: JQ217116-JQ217131) were reconstructed using Ovar-DRB1*0101 (DRB1*0101 allele of *Ovis* *aries*, GenBank accession number: Y07898) as outgroups. Orthologous sequences from *Macaca* *fascicularis* (*Mafa-DRB*), *Macaca* *mulatta* (*Mamu-DRB*), *Mandrillus* *sphinx* (*Masp-DRB*), *Pan* *troglodytes* (*Patr-DRB*), *Gorilla* *gorilla* (*Gogo-DRB*) and *Homo sapiens* (*HLA-DRB*) (GenBank accession numbers: KF880641, KF880647, M96121.1, M96122.1, DQ103723, DQ103724, DQ103725, DQ103732.1, AM086033, AM086040, AF031254, AF031271.1, FJ442950.1, DQ837166.1, EU934775.1, FN424202.1, HE800526.1, KR632831.1, HM580015.1, HM594301, FR717382, EF208835 and JQ579493.1) were included in the analysis. Best-fit models for nucleotide substitution (assuming a gamma distribution) were determined using the Akaike Information Criterion (AIC) in jmodeltest V2.1.3 [41]. Phylogenetic relationships were then estimated according to the Bayesian approach using mrbayes V3.0 [42], the maximum likelihood (ML) method using phyml V3.0 [43] and the maximum parsimony (MP) method using mega V7 [34], respectively. The reliability of each tree topology structure was carried out via 1000 bootstrap replications.

#### Selection pressure analysis

We calculated *ω* at all amino acid sites, ABS, and non-ABS in the exon 2 region in mega V7 [34] using the Nei-Gojobori method with the Jukes-Cantor correction [44] and 1000 bootstrap replicates to obtain standard errors. The putative ABS and non-ABS were both derived according to the structure of human *DRB* genes [45]. The statistical significance of the difference between *d*_N_ and *d*_S_ was tested using a *Z*-test implemented in mega V7 [34]. Evidence for natural selection was also obtained using the codeml program in paml V4.7 [46]. The heterogeneity of *ω* among codons was examined based on the maximum likelihood method. Six models (M0, M1a, M2a, M3, M7 and M8) allowing different selection intensity among sites were compared using likelihood-ratio tests in paml V4.7 [12, 46]. Posterior probabilities for site classes were calculated by the Bayes empirical Bayes (BEB) method in models M2a and M8.

## Results

### *DRB* allele assignment

We examined the variation of *DRB* genes of 122 *R. roxellana* monkeys from three populations (FP, NS and ZZ). Twenty clones were sequenced from both strands for each individual. Nineteen different *DRB* sequences were obtained with 270 bp, including 247 bp exon 2 and partial intron 1.

After performing alignments with Rhro-DRB*01–37 (GenBank accession numbers: JQ863322-JQ863358) [30], fourteen sequences were matched with Rhro-DRB*02, 03, 04, 05, 06, 07, 08, 09, 14, 16, 17, 18, 23 and *26,* respectively. Five novel *DRB* sequences were identified and labeled as Rhro-DRB*38, 39, 40, 41 and *42* (GenBank accession numbers: MF434639-MF434643) according to the nomenclature reported by Klein et al. [47]. Each of the new alleles identified in this study was present in at least three different PCRs, without stop codons, frame-shift mutations, or insertions/deletions. Rhro-DRB*23, first detected by Luo and Pan [30], lost three bases, which also resulted in one amino acid deletion. Two to four alleles were detected in each individual, indicating at least two loci were amplified, which was consistent with previous research [30]. According to the results of mhc-typer V1.0, 11 alleles were assigned to *Rhro-DRB1* and 8 alleles were assigned to *Rhro-DRB2* (*Rhro-DRB1*: 02, 03, 06, 09, 14, 17, 23, 38, 39, 41 and 42; *Rhro-DRB2*: 04, 05, 07, 08, 16, 18, 26 and 40).

### Genetic variation at *DRB* and microsatellites

DNA extracts were amplified at 19 microsatellite loci and two *DRB* genes. The characteristics of these loci are presented in Tables 2, 3 and 4. The number of alleles per microsatellite locus ranged from 3 to 6, with an average of 4.6. The number of alleles within the two *DRB* loci varied among the three study populations, ranging from 10 in FP to 14 in ZZ (Table 3). Some of these alleles were abundant (more than 30%) in all three populations (e.g., DRB*03 and DRB*04), whilst others were detected at low frequencies (less than 10%) and/or only in one population (e.g., DRB*18, *23*, *26*, *38*, *39*, *40*, *41* and *42*).

**Table 2:** Population genetic parameters for three populations estimated from microsatellite data

Population	No. of Loci	*k*	*A*_R_	PIC	*H*_O_	*H*_E_	*F*_IS_
FP	19	3.58	3.340	0.466	0.496	0.535	0.026
NS	19	3.74	3.606	0.451	0.506	0.512	−0.075
ZZ	19	3.68	3.457	0.461	0.514	0.534	0.038
All	19	4.63	3.839	0.520	0.531	0.581	–

*k* mean number of alleles for all loci, *A*_*R*_ allelic richness, *PIC* polymorphic information content, *H*_O_ and *H*_E_, observed and expected heterozygosity, respectively; *F*_IS_, inbreeding coefficient

**Table 3:** Allele frequencies of *MHC* in three *R. roxellana* populations in the Qinling Mountains

Locus	Allele	FP (6/4)^a^ *N* = 32	NS (7/6)^a^ *N* = 36	ZZ (8/6)^a^ *N* = 54	All (11/8)^a^ *N* = 122
*DRB1*	DRB*02	–	0.042	0.074	0.045
	DRB*03	0.625	0.583	0.370	0.500
	DRB*06	0.031	0.083	–	0.033
	DRB*09	0.031	0.083	0.213	0.127
	DRB*14	0.125	0.083	0.259	0.172
	DRB*17	0.125	0.083	0.046	0.078
	DRB*23	–	0.042	–	0.012
	DRB*38	–	–	0.019	0.008
	DRB*39	–	–	0.009	0.004
	DRB*41	0.063	–	–	0.016
	DRB*42	–	–	0.009	0.004
*DRB2*	DRB*04	0.667	0.583	0.519	0.576
	DRB*05	0.067	0.125	0.075	0.088
	DRB*07	–	–	0.038	0.017
	DRB*08	0.133	0.042	0.292	0.177
	DRB*16	0.133	0.125	0.066	0.101
	DRB*18	–	0.083	–	0.025
	DRB*26	–	0.042	–	0.013
	DRB*40	–	–	0.009	0.004

^a^Numbers in the front of and behind the slash represent the total allele number of *DRB1* and *DRB2*, respectively. N represents sample size in certain population

**Table 4:** Summary of *DRB* variation in the three *R. roxellana* populations

Locus	Pop	*N*	*k*	Nu	Co	ABS	Variable nucleotide positons	Variable codons	Variable ABS	PIS	Probability test	*P*_null_	*A*_R_	PIC	*A*_E_	*H*_E_	*H*_O_	Overall mean distance	*F*_IS_	Tajima’s *D* (*P*-value)
*DRB1*	FP	32	6	243	81	18	48	29	16	27	0.034 ± 0.000	−0.010	5.994	0.542	2.338	0.581	0.563	0.101 ± 0.015	0.033	0.643(*P* > 0.10)
	NS	36	7	243	81	18	54	29	16	26	0.170 ± 0.000	0.072	6.993	0.606	2.692	0.637	0.583	0.105 ± 0.015	0.086	0.519(*P* > 0.10)
	ZZ	54	8	243	81	18	54	30	16	41	0.038 ± 0.000	0.026	6.899	0.701	3.878	0.749	0.703	0.111 ± 0.014	0.061	1.062(*P* > 0.10)
	All	122	11	243	81	18	60	30	16	43	–	–	8.030	0.665	2.969	0.697	0.631	0.104 ± 0.015	–	0.977(*P* > 0.10)
*DRB2*	FP	30	4	243	81	18	37	19	8	8	0.109 ± 0.000	−0.056	4.000	0.479	2.064	0.524	0.533	0.092 ± 0.019	0.000	0.506(*P* > 0.10)
	NS	36	6	243	81	18	40	22	11	22	0.368 ± 0.001	0.065	5.993	0.589	2.618	0.627	0.583	0.082 ± 0.014	0.070	0.564(*P* > 0.10)
	ZZ	53	6	243	81	18	42	21	11	18	0.000 ± 0.000^a^	0.202	5.530	0.579	2.730	0.640	0.415	0.084 ± 0.016	0.353	0.326(*P* > 0.10)
	All	122	8	243	81	18	44	23	12	28	–	–	6.352	0.584	2.471	0.621	0.496	0.082 ± 0.014	–	0.608(*P* > 0.10)

*Pop* population, *N* number of genotyped samples, *k* number of alleles, *Nu* nucleotides, Co codons, *PIS* parsimony-informative sites; Probability test were apply for testing deviation from the Hardy-Weinberg equilibrium (HWE); *P*_null_, the frequency of null alleles; *A*_R_, allelic richness; PIC, polymorphic information content; *A*_E_, effective number of alleles; *H*_O_ and *H*_E_, observed and expected heterozygosity, respectively; the overall mean distance here was applied to the nucleotide sequence; *F*_IS_, inbreeding coefficient

^a^, Excess of homozygotes at *DRB2* locus for ZZ population

The *H*_O_ are generally lower than *H*_E_ in these three populations at both *DRB* loci, with the exception of the *DRB2* locus in the FP population (Table 4). For microsatellite loci, the *H*_O_ are all higher than *H*_E_ in three populations (Table 2). We only observed significant deviations from HWE (Bonferroni correction, *P* = 0.008, Table 4) and an excess of homozygotes at the *DRB2* locus in the ZZ population (*H*_O_ = 0.415; *H*_E_ = 0.640), in which a high frequency of null alleles was detected (*P*_null_ = 0.202). The levels of *H*_E_ were all above 0.5 among the three populations at two loci (*DRB1*: 0.581–0.749; *DRB2*: 0.524–0.640) and 19 microsatellite loci (0.512–0.535), indicating a high level of genetic diversity at both types of markers in all three populations. The values of PIC were generally over 0.5 for MHC genes, with the exception of the *DRB2* gene in the FP population (0.479), suggesting that other combinations had high polymorphism. For microsatellites, the values of PIC were all less than 0.5 (0.451–0.466), indicating that microsatellites had moderate polymorphism. Allelic richness (*A*_R_) also varied at two types of loci, with *DRB1* ranging from 5.994 to 6.993, *DRB2* ranging from 4.000 to 5.993, with microsatellites ranging from 3.340 to 3.606 (Tables 2 and 4).

The genetic diversity of *MHC* loci (average *H*_E_ = 0.626, PIC = 0.583) is higher than that of microsatellites (average *H*_E_ = 0.527, PIC = 0.459). Mann-Whitney *U* tests showed that the difference in *H*_E_ between two types of loci are not significantly different (*n*_1_ = 60, *n*_2_ = 6, *U* = 115.5, *P* = 0.194), whereas the PIC of *MHC* loci are significantly higher than that of microsatellites (*U* = 87.0, *P* = 0.049).

We identified 67 (27.1%) variable nucleotide positions in a 247 bp sequence. These sequences showed wide-ranging levels of divergence with an average of 24.7 (10%) nucleotide differences (minimum: 3 substitutions, maximum: 37 substitutions) among sequences. We detected 33 (40.1%) variable positions in the 81 amino acid sequence. The number of pairwise amino acid differences between sequences ranged from 2 to 22 with an average of 15.3 (18.9%). For locus-specific variation, we found 60 (24.7%) variable nucleotides for *DRB1* and 44 (18.1%) for *DRB2*, corresponding to 30 (37.0%) amino acid residue changes in *DRB1* and 23 (28.4%) changes in *DRB2* (Table 4; Additional file 1: Figure S1). The proportion of variable amino acids at the ABS for both loci exceeded 50% (16/18 in *DRB1* and 12/18 in *DRB2*), with the overall mean distance 0.104 ± 0.015 at the *DRB1* locus and 0.082 ± 0.014 at *DRB2*. Thus, both loci exhibit high levels of sequence divergence.

Among populations, the FP population had the lowest polymorphism at two *DRB* loci for most parameters (Table 4). The number of alleles (*DRB1*: 6; *DRB2*: 4), the variable nucleotide sites (*DRB1*: 48; *DRB2*: 37), the PIC (*DRB1*: 0.542; *DRB2*: 0.479) and *H*_E_ (*DRB1*: 0.581; *DRB2*: 0.524) in the FP population were all lower than in the other two populations (Table 4). However, the observed heterozygosity (*H*_O_ = 0.415) at *DRB2* locus in the ZZ population is lower than the other combinations (Table 4). We also found that the *MHC* allele frequencies of different populations were less differentiated than microsatellite allele frequencies (Table 5). *F*_*ST*_ values of adaptive *MHC* genes and neutral microsatellites among the three study populations are shown in Table 5.

**Table 5:** Summary of population differential (*F*_ST_) in the three *R. roxellana* populations

Locus	Population	FP	NS
*MHC*	NS	0.002	
	ZZ	0.051	0.053
Microsatellite	NS	0.108	
	ZZ	0.132	0.108

### Positive selection

The selection parameter *ω* (*d*_N_/*d*_S_, the rate of non-synonymous substitutions/synonymous substitutions) was calculated for the ABS, non-ABS and all amino acid positions (Table 6). For the ABS sites across all alleles, *ω* was significantly greater than one (*ω* = 6.807, *P* = 0.000), indicating that there was a strong positive selection at these sites in the *Rhro-DRB* sequences. For the non-ABS sites, *ω* was less than one (*ω* = 0.505, *P* = 0.013), suggesting negative/purifying selection at the non-ABS sites.

**Table 6:** Rate of non-synonymous substitutions (*d*_N_) and synonymous substitutions (*d*_S_)

Sites	*d*_N_	*d*_S_	*ω*	*P*
ABS	0.388 ± 0.071	0.057 ± 0.038	6.807	0.000
Non-ABS	0.053 ± 0.013	0.105 ± 0.034	0.505	0.013
All	0.111 ± 0.019	0.094 ± 0.026	1.181	0.597

Amino acid residues under significant positive selection were also found with paml V4.7 (Table 7) using the maximum likelihood method. Various codon evolutionary models were compared using codeml program in paml V4.7 [46]. With regard to the Akaike Information Criterion (AIC) values, models integrating positive selection (M2a, M3, and M8) matched *MHC* better than the other models (Tables 7 and 8). Under models M2a and M8, two sites (11F and 13S) were exposed to significant selection. Under model M3, 32 sites were identified, in which 27 sites (9E, 10Q, 11F, 13S, 26Y, 28Q, 30Y, 31F, 32Y, 37Y, 38 V, 47F, 49A, 56P, 57 V, 60 N, 61F, 64Q, 67F, 70Q, 71R, 72R, 74Q, 77 N, 78Y, 84G and 86 V) including both 11F and 13S, showed significant selection pressure (Table 7). Moreover, most of these sites were associated with a peptide and/or a T-cell receptor (TCR) (Additional file 1: Figure S1). Collectively, these results indicate that in *R. roxellana*, most of the positive selection we identified occurred at functionally important sites.

**Table 7:** Results of maximum likelihood models of the *Rhro-DRB* sequences

Model	*k*	*l*	Parameters estimates	Positively selected sites
M0 (one ratio)	1	− 1830.09	*ω* = 0.559	None
M1a (nearly neutral)	1	− 1690.63	*p*_0_ = 0.603 (*p*_1_ = 0.397)	Not allowed
M2a (positive selection)	3	− 1653.33	*p*_0_ = 0.576, *p*_1_ = 0.400 (*p*_2_ = 0.025), *ω*_2_ = 12.820	**11F**, **13S**
M3 (discrete)	5	− 1651.07	*p*_0_ = 0.602, *p*_1_ = 0.374 (*p*_2_ = 0.025), *ω*_0_ = 0.035, *ω*_1_ = 1.496, *ω*_2_ = 16.973	6R, **9E**, **10Q**, **11F**, **13S**, 15C, 25R, **26Y**, **28Q**, **30Y**, **31F**, **32Y**, **37Y**, **38 V**, **47F**, *49A*, **56P**, **57 V**, **60 N**, **61F**, 63S, **64Q**, **67F**, **70Q**, **71R**, *72R*, **74Q**, **77 N**, **78Y**, **84G**, 85 V, **86 V**
M7 (beta)	2	− 1686.84	*p* = 0.079, *q* = 0.125	Not allowed
M8 (beta and omega)	4	−1651.44	*p*_0_ = 0.975 (*p*_1_ = 0.025), *p* = 0.019, *q* = 0.035, *ω* = 11.880	**11F**, **13S**

*K* represents the number of parameters in the *ω* distribution, *ω* is the parameter of selection, *p*_n_ is the proportion of sites falling into the *ω*_n_ sites class. For models M7 and M8, *p* and *q* are the shape parameters of the β function. Sites inferred under selection at the 99% level are listed in bold, and those inferred at the 95% level are shown in italics

**Table 8:** Likelihood-ratio test of codon evolution for exon 2 sequences at *Rhro-DRB* loci

Models compared	d*f*	Test statistic	Significance (*P*)
M2a vs. M1a	2	74.6	< 0.001
M3 vs. M0	4	358.04	< 0.001
M8 vs. M7	2	70.8	< 0.001

The values for Tajima’s *D* statistics for the two *DRB* loci of all three populations were all positive but did not differ significantly from the equilibrium neutral expectation (Table 4).

### Trans-species evolution

**Fig. 2:**
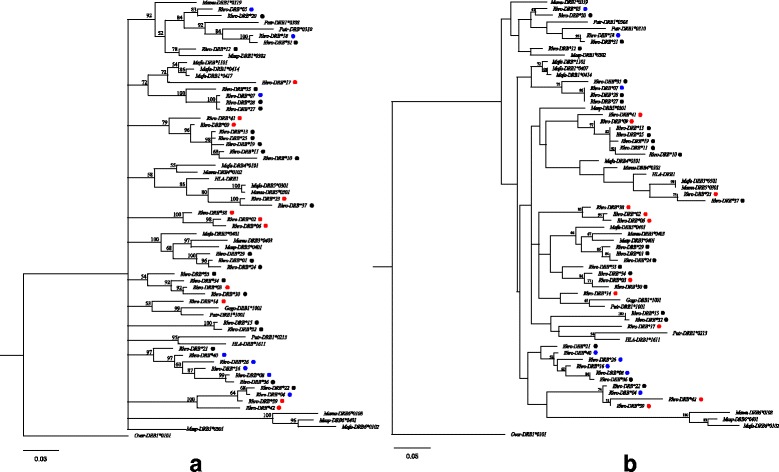
Phylogenetic relationships of *Rhro-DRB* alleles conducted using Bayesian approach (**a**) and maximum likelihood method (**b**). Orthologous sequences from *Ovis* *aries* (Ovar-DRB1*0101)*,* *Macaca* *fascicularis* (*Mafa-DRB*), *Macaca* *mulatta* (*Mamu-DRB*), *Mandrillus* *sphinx* (*Masp-DRB*), *Pan* *troglodytes* (*Patr-DRB*), *Gorilla* *gorilla* (*Gogo-DRB*) and *Homo sapiens* (*HLA-DRB*) were include in the analysis. Values on the branch are represented for the posterior probability of the Bayesian tree and the support rate of the ML tree. Sequences labeled with solid circles are *DRB* alleles from *R. roxellana*. Colored circles indicate sequences detected in the three study populations. Red ones indication *Rhro-DRB1* alleles, while blue ones indicate *Rhro-DRB2* alleles

In order to investigate the evolutionary relationships of *DRB* sequences among *R. roxellana* and other primates, Bayesian, ML and MP phylogenetic trees were constructed (Fig. 2; Additional file 2: Figure S2). Ovar-DRB1*0101 was selected as the outgroup, and only bootstrap values/posterior probabilities greater than 50% were included. This showed that consistent with trans-species evolution, the allelic relationships were inconsistent with the species relationships, and alleles from different species intermixed with each other. No clear clade for *Rhro-DRB* was identified in the phylogenetic tree.

## Discussion

### Patterns of the *Rhro-DRB* diversity

We measured the genetic variation of the *Rhro-DRB* genes of 122 individuals from three *R. roxellana* populations in the Qinling Mountains, and found 19 different *DRB* alleles, of which five alleles were novel. Rhro-DRB*23, which lost three bases and resulted in one amino acid deletion, is considered a functional allele [30]. After performing BLAST on the NCBI website (https://blast.ncbi.nlm.nih.gov/Blast.cgi), we found several homologous sequences in other primate species which have lost the same three bases as has Rhro-DRB*23. Many of these sequences were expressional mRNA sequences, such as *Mafa-DRB* (GenBank accession numbers: HM580023, JQ579479, LT746055 and LT707677); *Mamu-DRB* (GenBank accession number: KF880635.1); *Mane-DRB* (*Macaca* *nemestrina*, GenBank accession number: JQ693980); *Paur-DRB* (*Papio* *ursinus*, GenBank accession number: DQ339731) and *Caja-DRB* (*Callithrix* *jacchus*, GenBank accession numbers: LN906590, LN906591 and LT908516). We thus conclude that Rhro-DRB*23 is functional and was present in the common ancestor of all species in which it is currently present.

Two to four alleles were detected within each individual, indicating at least two *DRB* loci were sequenced in this study. In addition, a high similarity of alleles between two loci were found, suggesting that gene duplication plays a role in increasing copy numbers of *DRB* genes. Gene duplication among *MHC* genes has also been observed in other mammals [48–50]. Three of 19 alleles (16%) were found only once among our samples, together with the detection of many novel alleles (five in 19). This suggests that there may be rapidly evolving loci within this species consistent with *MHC class II* genes in mammals that are found to have high duplication rates [51]. Given the high frequency (more than 30%) of the alleles DRB*03 and *04* among all three *R. roxellana* populations used for this study, it is possible that these alleles may be subject to particular selective pressures that allow the sequences to persist longer than expected under neutrality [52].

The diversity of *MHC class II* genes of several primate species has been investigated over the past two decades, especially those in the rhesus macaque (*Macaca mulatta*) [53, 54]. This is an important model species in preclinical transplantation research and for the study of chronic and infectious diseases. Much of those research focused on the *Mamu-DRB* haplotype, and compared with humans, revealed high levels of polymorphism at the *Mamu-DRB* region configurations [53]. More recently, extensive research on non-model primate species have evaluated the adaptive nature of this genetic diversity. For example, 16 different *DRB* sequences were detected in 30 chacma baboons (*Papio ursinus*) [55]. These exhibited wide-ranging divergence based on 92 (36.5%) variable nucleotides in a 252 bp sequence, and 40 (47.6%) variable sites in a 84 amino acid sequence [55]. The mouse lemur (*Microcebus murinus*) also shows high levels of sequence divergence. In this species, 12 different *DRB* alleles were found in 145 individuals, with 58 (33.9%) variable positions in the 171 bp sequence and 29 (50.9%) variable positions out of 57 amino acids [56]. Schwensow et al. [57] also found much genetic variability in fat-tailed dwarf lemurs (*Cheirogaleus medius*), with 50 different *DRB* alleles differing at one to 42 positions from each other, and 33 (57.9%) out of 57 amino acid positions being variable. In this study, we found that the *DRB* sequence divergence in the three study *R. roxellana* populations is relatively low compared with *Ma. mulatta*, *P. ursinus*, *Mi. murinus* and *C. medius.* We identified 67 (27.1%) variable nucleotide positions with an average of 24.7 (10%) differences among sequences corresponding to 33 (40.1%) variable amino acid positions with an average of 15.3 (18.9%) differences among sequences were detected. The proportion of variable amino acids at the ABS for both loci is more than 50% (16/18 in *DRB1* and 12/18 in *DRB2*), meaning that most residual changes occurred in functionally important regions. This is similar to other *MHC* loci not only in other primates species [57, 58], but also in other vertebrate species, such as the mummichog (fish) (*Fundulus heteroclitus*: [59]), the red-eyed tree frog (*Agalychnis callidryas*: [60]), the lesser kestrel (*Falco naumanni*: [61]) and the giant panda (*Ailuropoda melanoleuca*: [62]).

The PIC and *H*_E_ of two *DRB* loci both exceed 0.5 (*DRB1*: PIC = 0.665, *H*_E_ = 0.697; *DRB2*: PIC = 0.584, *H*_E_ = 0.621), indicating a high genetic diversity for *DRB* genes, which is congruent with results obtained for two other *MHC* genes (*DQA1*: PIC = 0.662, *H*_E_ = 0.715; and *DQB1*: PIC = 0.658, *H*_E_ = 0.713) [63]. In fact, our observed polymorphism is likely to underestimate the variation across the entire exon 2, given that our *DRB* sequences covered only 247 bp in the exon.

Among populations, the FP population had the lowest polymorphism for *DRB* genes in most parameters except *H*_O_ (Table 4). The lowest *H*_O_ was in the ZZ population. The observed heterozygosity can be influenced by many factors, including inbreeding, selection, random effect, and null alleles. Inbreeding can result from non-random mating, including mating between closely related individuals, and pervasive inbreeding (due to genetic drift in a small or subdivided population) [64]. However, we found inbreeding coefficients at 19 microsatellites ranged from − 0.075 to 0.038, indicating there is little or no effect of inbreeding in each of the three study populations [65]. The observed degree of heterozygosity may also be affected by selection, which relies on fitness variation among individuals of different genotypes through both differential survival and differential reproduction (e.g. though non-random mating) [66, 67]. The low overall inbreeding coefficients in each population estimated from microsatellites suggest that non-random mating has little effect. Selection is thus unlikely to be the sole cause of the observed heterozygote deficiencies. Any random effects fail to explain the excess of homozygotes - our testing the hypothesis that *H*_O_ is equal to *H*_E_ and any difference is due to random error being rejected (*P* = 0.0007). We suggest that the most parsimonoious explanation for the excess of homozygotes in the ZZ population is the presence of null alleles. These are alleles that cannot be amplified, usually due to the mutations at the primers binding sites [68]. The frequency of null alleles at the *DRB2* locus in the ZZ population is the highest out of the three study populations (*P*_null_ = 0.202), which increases the levels of observed homozygosity. Hence, the inbreeding coefficient in the ZZ population is over-estimated, and the genotypic frequencies deviate significantly to those expected from the HWE at the *DRB2* locus (Table 4).

### Evidence for balancing selection

Key aspects of a species’ adaptations to challenging environments are likely to have been pathogen-mediated [69]. Immunity-related *MHC* is an ideal model gene family for studying host-pathogen coevolution [9]. Many wild populations have suffered from a reduction in *MHC* diversity after past population decline [19, 70, 71]. However, in this study, we found high levels of *DRB* diversity in Qinling *R. roxellana* populations that suffered a rapid reduction in numbers and severe population fragmentation over the past several decades. We found several lines of evidence that suggest balancing selection has been acting on *DRB* variation in this species.

First, in all three populations, population genetics analysis showed that neutral variation is relatively lower than variation for adaptive *MHC* genes. The different diversity patterns of *MHC* and neutral genes suggest that selection rather than neutral demographic processes (such as genetic drift, bottleneck and/or founder effect) results in a high level of genetic diversity for adaptive *MHC* genes [1, 9, 17]. According to Huang et al. [28], although the Qinling *R. roxellana* populations suffered a rapid reduction in population size [23], there is no evidence of a past genetic bottleneck. Pan et al. [27] found much polymorphism in *R. roxellana* at the mitochondrial control region (D-loop), suggesting that any influence of a founder effect on genetic diversity is weak. Despite the strength of influence of genetic drift, past bottlenecks and/or founder effects in our study populations, these factors will reduce genetic diversity. Similar patterns of high variability of *MHC* genes coupled with relatively low microsatellite variability occurs in natural populations of other vertebrate species. For example, the house finch (*Carpodacus mexicanus*), a species that has experienced past population decline due to a disease epidemic, has high multi-locus *MHC* diversity but relatively moderate levels of variability at microsatellites [72]. A more extreme example is the San Nicholas Island fox (*Urocyon littoralis dickeyi*), a critically endangered species that has high MHC heterozygosity but has little if any microsatellite genetic variation [73].

Second, we found that the *MHC* allele frequencies of the three populations were less differentiated than the microsatellite allele frequencies (Table 5). This is as predicted for a gene under balancing selection or one closely linked to such a locus [73]. In a relatively small geographic region, where populations experience similar pathogen mediated selective regimes, i.e. homogenous selection pressure, balancing selection tends to decrease the levels of among-population variation [10, 74]. Therefore, the low differentiation among *R. roxellana* populations in the Qinling Mountains might be a result of homogenous balancing selection.

Third, positive Tajima’s *D* values at two *DRB* loci (Table 4) indicated that the number of alleles at intermediate frequency was higher than expected, possibly as a result of balancing selection and/or rapid population contact [40]. However, the Tajima’s *D* statistics do not differ significantly so we cannot reject the null hypothesis that the *DRB* gene is under neutral selection. Even so, there is additional evidence supporting positive selection. Nucleotide sites under positive selection are expected to accumulate more non-synonymous than synonymous substitutions, eventually bringing about amino acid changes and corresponding functional changes in MHC proteins [60]. Such adaptive evolutionary processes, possibly due to pathogen-mediated balancing selection, should be evident at the ABS [60]. According to the proposed criteria, *R. roxellana DRB* genes showed evidence of positive selection for diversification. Positive selection acted only on ABS codons, with a significantly increased level of non-synonymous substitutions, whereas non-ABS codons exhibited significant negative/purifying selection. Rates of non-synonymous substitutions were 7.32 times higher in the ABS than the non-ABS. This suggests that the polymorphism due to positive selection, in functionally important regions of the MHC molecule, allows *R. roxellana* to respond to a wider range of pathogens.

Additionally, our six random sites model analysis in paml V4.7 [46] revealed the existence of positive selection in the maximum likelihood method. Our results suggested that the models including selection (M2a, M3 and M8) matched *DRB* alleles better than those without selection (Tables 7 and 8). Under the M2a and M8 models, the same two ABS sites (11F and 13S) were exposed to significant selection, whilst under the M3 model, 32 sites including most ABS sites (17 of 18) were detected as being under positive selection. Our results are in accordance with substitutions often occurring within a functionally important domain [60].

Further evidence for balancing selection was provided by our result showing trans-species evolution of the *DRB* alleles. Pathogen mediated balancing selection can result in allele retention among species for long evolutionary time periods, resulting in similar or even identical alleles being shared among extant species [18]. Such balancing selection gives rise to patterns of gene trees for the *MHC* that differ from species relationships, termed ‘trans-species evolution’ [75]. Evidence for trans-species evolution for the *MHC* gene complex has been previously found in a variety of vertebrate taxa (fishes: [76]; amphibians: [60]; reptiles: [77]; birds: [78]; mammals: [48]). In this study, we present clear phylogenetic evidence of trans-species evolution of *DRB* sequences across *R. roxellana*, *Macaca fascicularis*, *Macaca mulatta*, *Mandrillus sphinx,* *Pan* *troglodytes* and even *Homo sapiens* (Fig. 2; Additional file 2: Figure S2). This suggests that due to balancing selection, some allelic lineages have been retained over long evolutionary time periods and certain alleles that are shared among species are older than the diversification time of species or even families.

## Conclusions

Many wild species are threatened by a dramatic reduction in and fragmentation of habitat, geographic isolation, small and declining populations and a decrease in genetic diversity [24, 79–82]. Understanding the patterns of adaptive diversity of threatened species is crucial. *MHC* genes play an important role in adaptive immunology, and are ideal markers to study adaptive evolution [18]. Many wild populations have suffered from a reduction in *MHC* diversity after population decline [70]. However, in this study, we found high levels of *DRB* diversity in three *R. roxellana* populations that have suffered a rapid reduction in numbers and severe population fragmentation over the past several decades. We also found evidence of pathogen-mediated balancing selection, which is likely to have contributed to maintaining *MHC* polymorphism over time. Our study adds to information on *MHC* genes and may assist in developing effective management strategies for *R. roxellana*. Our new data furthers our understanding of the role pathogen-mediated balancing selection has in maintaining variation in *MHC* genes in small and fragmented populations of free-ranging vertebrates.

## Additional files

Additional file 1: **Figure S1.** Sequence alignments of the deduced amino acid sequences for exon 2 of *Rhro-DRB* sequences. *DRB* sequences were taken from this study and previously published research (Luo and Pan 2013) and were included in sequence alignments. Dots indicate identity with the first sequence.”+” and “*“on the alignment represents putative ABS and sites contact to TCR, respectively. The putative ABS and sites contact to TCR were both derived according to the structure of human *DRB* genes (Reche and Reinherz, 2003). (PDF 308 kb)

Additional file 2: **Figure S2.** Phylogenetic tree of *Rhro-DRB* alleles using the maximum parsimony method. Orthologous sequences from *Ovis* *aries* (Ovar-DRB1*0101)*,* *Macaca* *fascicularis* (*Mafa-DRB*), *Macaca* *mulatta* (*Mamu-DRB*), *Mandrillus* *sphinx* (*Masp-DRB*), *Pan* *troglodytes* (*Patr-DRB*), *Gorilla* *gorilla* (*Gogo-DRB*) and *Homo sapiens* (*HLA-DRB*) were include in the analysis. Values on the branch are represented for the support rate of the MP tree. Sequences labeled with solid circles are *DRB* alleles from *R. roxellana*. Colored circles indicate sequences detected in the three study populations. Red ones indication *Rhro-DRB1* alleles, while blue ones indicate *Rhro-DRB2* alleles. (PDF 183 kb)

## Abbreviations

*MHC*: *major histocompatibility complex* ABS Antigen binding sites TCR T-cell receptor FP Foping NS Ningshan ZZ Zhouzhi ML Maximum likelihood MP Maximum parsimony
