# Supplementary material for: High polymorphism in MHC-DRB genes in golden snub-nosed monkeys reveals balancing selection in small, isolated populations
Source: BMC Evol Biol. 2018 Mar 13;18:29. doi: 10.1186/s12862-018-1148-7 (PMC5851093; doi:10.1186/s12862-018-1148-7)
Supplement: Supplementary file 1 — Figure S1. Sequence alignments of the deduced amino acid sequences for exon 2 of Rhro-DRB sequences. DRB sequences were taken from this study and previously published research (Luo and Pan 2013) and were included in sequence alignments. Dots indicate identity with the first sequence.”+” and “*“on the alignment represents putative ABS and sites contact to TCR, respectively. The putative ABS and sites contact to TCR were both derived according to the structure of human DRB genes (Reche and Reinherz, 2003). (PDF 308 kb) [file 12862_2018_1148_MOESM1_ESM.pdf]

```
                  10         20         30         40         50         60         70         80
               ....| .........| .........| .........| .........| .........| .........| .........| ......
                                                                          *      ** ** ***  **    *
                  +  + +             + + +       ++         +          +    +     +    +  +   +    +  ++
Rhro-DRB*01    RFLEQ FKSECHFFNG TERVRYLQRY FYNQEEYVRF DSDVGEFRAV TELGRPVAEN FNSQKDFLEQ RRAQVDNYCR HNYGVV
Rhro-DRB*02    ..... V.F....... .....F.E.R VH.....A.. ........E. ......D..Y W.G...L..D ...S...... ......
Rhro-DRB*03    ..... V.Y....... .....L.E.H ......FL.. ......Y... .......... W..RR.Y... ...A..T... ......
Rhro-DRB*04    ..... A......... .....F.D.. IH.R...... .......... ......D..Y W..R..I..R A..A..TV.. ...R.D
Rhro-DRB*05    ....Y ST........ .....F.D.. .......... ......Y... ......D... W.....I..D ...A..TF.. ...R.G
Rhro-DRB*06    ..... V.F....... .....F.E.R VH.....A.. ........E. ......D..Y W.G...L..D ...A...... .....G
Rhro-DRB*07    ..... V.H....... .....F.D.. IS....N... ......Y... ......D... W.....V... E..A..T... ...R.G
Rhro-DRB*08    ...W. P.R....... .....F.D.. .......... ......Y.E. .....RS..Y W.....I..R A..A..T... ......
Rhro-DRB*09    ...Q. G.A....... ....Q..E.. I......... ......Y... ......D... W.....L..D ...S..T... ......
Rhro-DRB*10    ...Q. G.A....... ....Q..E.. I.....LL.. .......... .......... W.....I..D. ..R..I-.. ......
Rhro-DRB*11    ...Q. G.A....... ....Q..E.H I.....FL.. .......... .......... W.....L..D. ..S..T... ......
Rhro-DRB*12    ....Y ST........ .....F.D.. .......... .......... ......D..Y W.....L..RK ..E..TF.. ...R.G
Rhro-DRB*13    ...Q. G.A....... ....Q..E.H I.....F... .......... .......... W.....L..D. ..S..T... ......
Rhro-DRB*14    ..... A.C....... .....F.E.R VH.R...A.. ......Y... ......D.KY W.....L..R. ..A..TF.. ......
Rhro-DRB*15    ...W. Y.P....... .....L.E.. ......N... ......Y... ......D... W..RR.L.... ..E..TV.. ...RI.
Rhro-DRB*16    ..... V.H....... .....F.D.. .......... ......Y.E. .....RS..Y W.....I..RA ..A..T... ......
Rhro-DRB*17    ...W. P.R....... .....F.D.. ......F... ......Y... .......... L..R......A L.A...... .....A
Rhro-DRB*18    ....Y TT........ .....F.D.. .......... .......... .....RS..Y W........D. ..S..T... ......
Rhro-DRB*19    ...Q. G.A.Y..... ....Q..E.H I.....FA.. .......... .......... W.....L..D. ..S..T... ......
Rhro-DRB*20    ....Y ST........ .....F.D.. .......... ......Y.E. ......D..Y W..R..I..DK ..A..TV.. .....G
Rhro-DRB*21    ..... A......... .....F.D.. .......... .......... .....RS..Y W.....L..RK Q.A..T... ......
Rhro-DRB*22    ..... A......... .....F.D.. IH.R...... .......... ......D..Y W..R..I..RA ..A..TV.. ...R.D
Rhro-DRB*23    ...K. D.H....... .......H.D I.....DL.. ......Y... ......D..Y W.......... ..E..K-.. ......
Rhro-DRB*24    ..... .......... .......... .......A.. .......... .......... ........... ......... ..C...
Rhro-DRB*25    ...Q. G.A....... ....Q..E.H I.....F... .......... .......... W.....L..D. ..S..T... ......
Rhro-DRB*26    ....Y ST........ .....F.D.. .......... ........E. .....RS..Y G.....I..RA ..A..T... ......
Rhro-DRB*27    ..... V.H....... .....F.D.. IS....N... ......Y... ......D... W.....V...E ..A..T... ...R.G
Rhro-DRB*28    ..... V.H....... .....F.D.. IS....N... ......Y... ......D... W.....V...E ..A..T... ...R.G
Rhro-DRB*29    ..... A......... .......... .......... .......... .......... ........... ......... ......
Rhro-DRB*30    ..... V.Y....... .....L.E.H ......FL.. ......Y... .......... W..RR.Y.... ..A..T... ...R.G
Rhro-DRB*31    ....Y TT........ .....F.D.. .......... .......... .....RS..Y W.G......D. ..S..T... ......
Rhro-DRB*32    .S.W. Y.P....... .....L.E.. ......N... ......Y... ......D... W..RR.L.... ..E..TV.. ...RI.
Rhro-DRB*33    ..... A.Y....... .....L.E.H ......FL.. .......... .....RS..Y W.......... ..A..T... ......
Rhro-DRB*34    ..... A......... .....L.E.H ......FL.. ......Y... .......... W..RR.Y.... ..A..T... ......
Rhro-DRB*35    ..... V.H....... .....F.D.. IS....N... ......Y... ......D... W.....L..D. ..S..T... ......
Rhro-DRB*36    P..W. P.R....... .....F.D.. .......... ......Y.E. .....RS..Y W.....I..RA ..A..T... ......
Rhro-DRB*37    ...K. D.H....... .......H.D I.....DL.. ......Y... ......D..Y W.....I..D. ..A..T... ......
Rhro-DRB*38    ..... V.F....... .....F.E.H ......FL.. ......Y.E. ......D..Y W.G...L..D. ..A...... ......
Rhro-DRB*39    ..... A......... .....F.D.. IH.R..FL.. .......... ......D..Y W..R..I..RA ..A..TV.. ...R.D
Rhro-DRB*40    ..... A......... .....F.D.. .......... ......Y.E. .....RS..Y W.....I..RA ..A..T... ......
Rhro-DRB*41    ...Q. G.A....... ....Q..E.. I......... ......Y... ......D... W.....L...A L.A...... .....A
Rhro-DRB*42    ...Q. G.A....... ....Q..E.. I.....FL.. .......... ......D..Y W..R..I..RA ..A..TV.. ...R.D
```
