# Supplementary figures and images for: High polymorphism in MHC-DRB genes in golden snub-nosed monkeys reveals balancing selection in small, isolated populations
Source: BMC Evol Biol. 2018 Mar 13;18:29. doi: 10.1186/s12862-018-1148-7 (PMC5851093; doi:10.1186/s12862-018-1148-7)

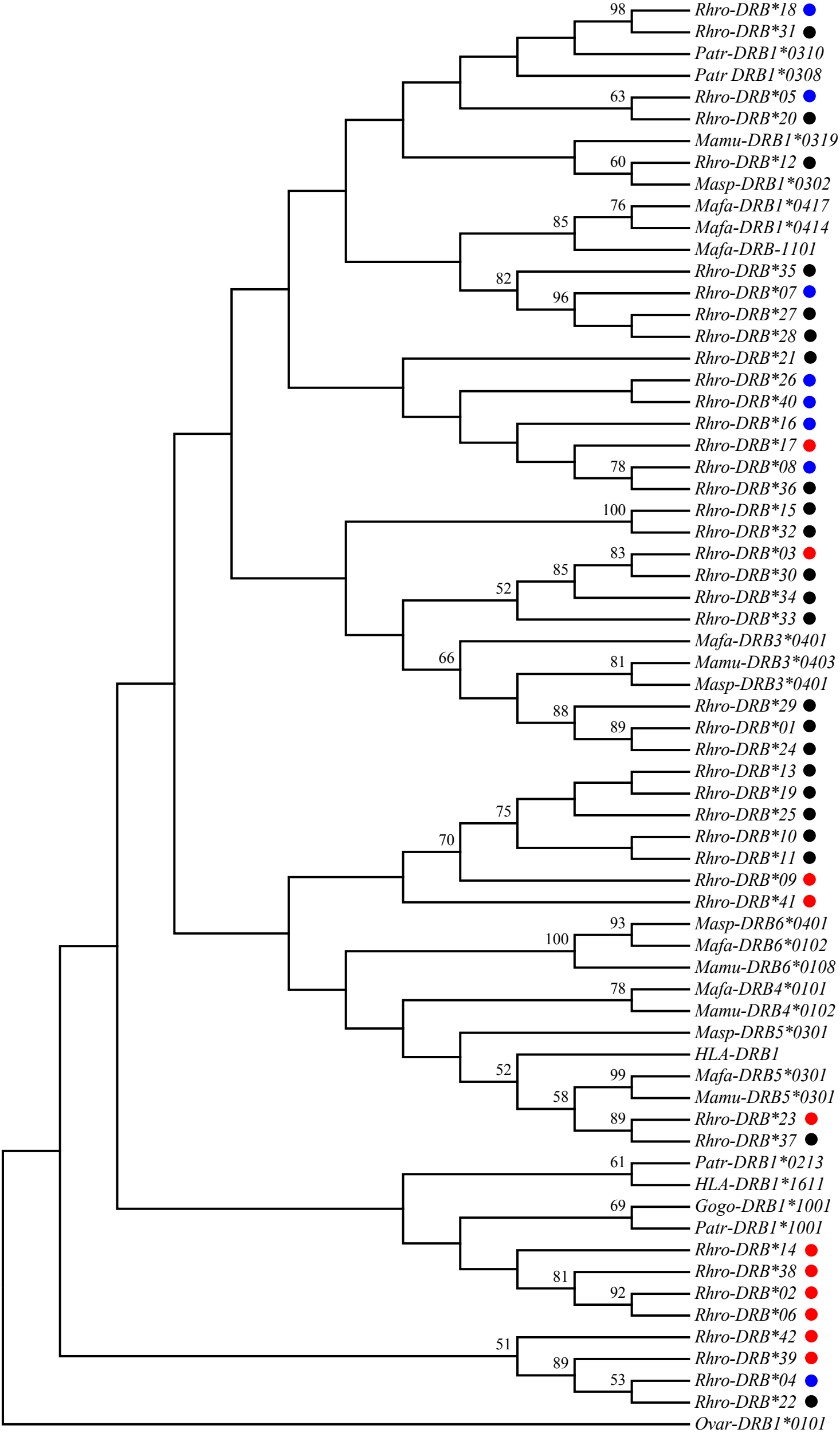

Supplement: Supplementary file 2 — Figure S2. Phylogenetic tree of Rhro-DRB alleles using the maximum parsimony method. Orthologous sequences from Ovis aries (Ovar-DRB1*0101), Macaca fascicularis (Mafa-DRB), Macaca mulatta (Mamu-DRB), Mandrillus sphinx (Masp-DRB), Pan troglodytes (Patr-DRB), Gorilla gorilla (Gogo-DRB) and Homo sapiens (HLA-DRB) were include in the analysis. Values on the branch are represented for the support rate of the MP tree. Sequences labeled with solid circles are DRB alleles from R. roxellana. Colored circles indicate sequences detected in the three study populations. Red ones indication Rhro-DRB1 alleles, while blue ones indicate Rhro-DRB2 alleles. (PDF 183 kb) [file 12862_2018_1148_MOESM2_ESM.pdf]
